# Normal Immune Function in a Newborn With Early Identification of a *SAMD9* Mutation Presenting With Growth Restriction, Thrombocytopenia, and Primary Adrenal Insufficiency

**DOI:** 10.1002/ccr3.71453

**Published:** 2025-11-18

**Authors:** Kevin MingJie Gao, Qiu Yu Judy Huang, Polly Huang, Anum Muzaffar, Ramin Beheshti, Robert Wood, Jennifer Dantzer, Maria J. Gutierrez

**Affiliations:** ^1^ Division of Innate Immunity, Department of Medicine UMass Chan Medical School Worcester Massachusetts USA; ^2^ Division of Rheumatology, Department of Medicine UMass Chan Medical School Worcester Massachusetts USA; ^3^ Department of Biochemistry and Molecular Biotechnology UMass Chan Medical School Worcester Massachusetts USA; ^4^ Division of Pediatric Allergy, Immunology, and Rheumatology, Department of Pediatrics Johns Hopkins University Baltimore Maryland USA

**Keywords:** inherited bone marrow failure, monogenic disorder, primary adrenal insufficiency, primary immune deficiency

## Abstract

MIRAGE syndrome is a multi‐organ disease caused by gain‐of‐function (GOF) mutations in the viral restriction factor SAMD9. Herein, we present the case of a 32‐week pre‐term female neonate with severe intrauterine growth restriction, primary adrenal insufficiency, and persistent thrombocytopenia. A rapid trio‐whole exome sequencing at 23‐days of age found a de novo SAMD9 G1048R mutation, consistent with a diagnosis of MIRAGE syndrome, and at this time was not found to have evidence of immune abnormalities or hematologic malignancy. Analysis of predicted tertiary structures from our patient’s SAMD9 G1048R mutation demonstrated structural similarities to known SAMD9 GOF variants. Absence of immunologic and oncologic manifestations in this patient may relate to early identification and reflect low SAMD9 expression during the neonatal window. Risk for developing immune deficiency and malignancy may continue to increase as this patient grows older, thus requiring close outpatient surveillance to mitigate future risk of these complications.

## Graphical abstract

A *de novo SAMD9* variant G1048R is associated with MIRAGE syndrome symptoms in a neonate. *SAMD9* G1048R's predicted tertiary structure has similarity to known MIRAGE variants. *SAMD9* expression is increased in pediatric viral infection. Neonatal MIRAGE may lack immune deficiency and myelodysplastic syndrome.

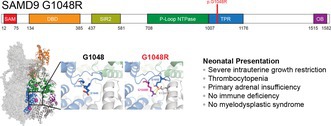

**Abbreviations**
ACTH: Adrenocorticotropic hormone
CBC: Complete Blood Count
CMV: Cytomegalovirus
CPAP: Continuous positive airway pressure
CRP: C‐reactive protein
CXR: Chest X Ray
FHT: Fetal heart tracing
FISH: Fluorescence in situ hybridization
GOF: Gain of function
IBMF: Inherited bone marrow failure
IFN: Interferon
IRE: Interferon response element
IUGR: Intrauterine growth restriction
IVIG: Intravenous immunoglobulin
MDS: Myelodysplastic Syndrome
NICU: Neonatal Intensive Care Unit
NK: Natural Killer
PAMP: Pathogen Associated Molecular Pattern
SNP: Single nucleotide polymorphism
SVT: Supraventricular tachycardia
TREC: T‐cell Receptor Excision Circle
VUS: Variant of unknown significance
WES: Whole exome sequencing
WT: Wild‐type

## 1 Introduction

**FIGURE 1:**
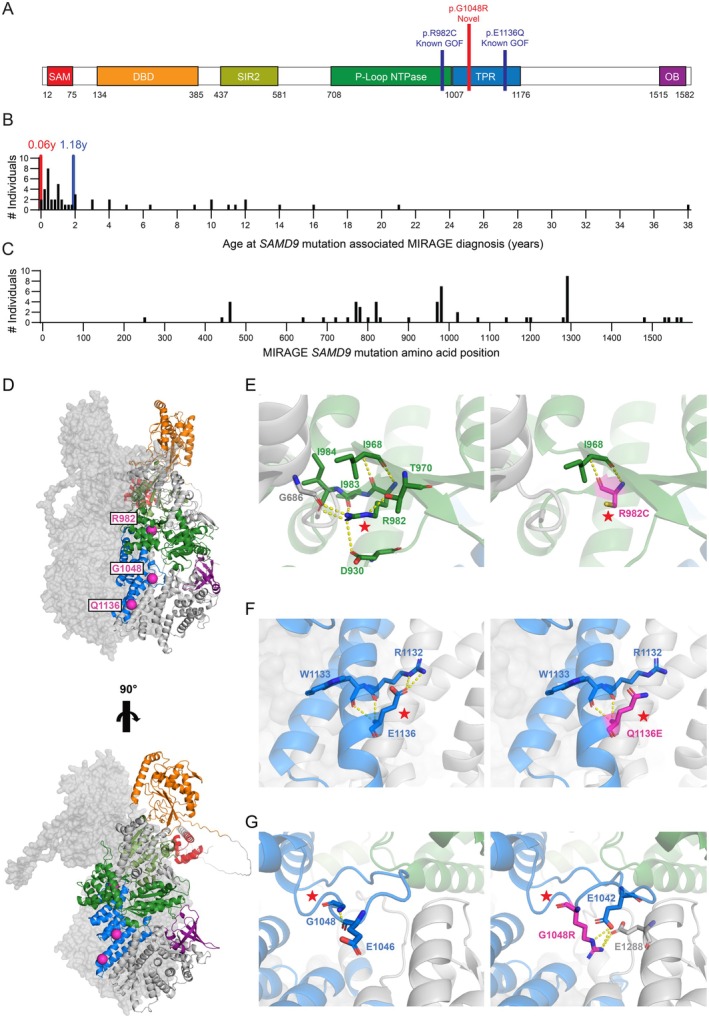
The *SAMD9* G1048 mutation is proximal to *SAMD9* gain‐of‐function variants that cause MIRAGE and shares features of hydrogen bond disruptions. (A) Diagram of *SAMD9* gene with the following predicted [1] or structurally confirmed [3, 4] domains indicated as colored boxes: SAM (red), DBD (orange), SIR2 (yellow), P‐Loop NTPase (green), TPR (blue), OB (purple). Start and ending amino acid positions for each domain are labeled. Two known MIRAGE causing *SAMD9* mutations are annotated with lines—p.R982C and p.E1136Q (blue), as well as the patient's novel mutation p.G1048R (red). (B) Histogram of age at genetic diagnosis of MIRAGE syndrome associated with *SAMD9* mutations generated by cross referencing case reports [5]. Age at death is shown if patient was diagnosed posthumously. A total of *n* = 31 patients were included. Each bar represents a bin of 0.2 years. A blue dotted line marks the median age of genetic testing for MIRAGE syndrome (1.18 years), the red dotted line marks the age of the patient in this case report at genetic testing (0.06 years). (C) Histogram of amino acid locations of MIRAGE syndrome causing *SAMD9* mutations obtained from a curated database with *n* = 56 patients [5]. Each bar represents a bin of 10 amino acid positions. (D) Structure of WT *SAMD9* as a dimer was predicted using AlphaFold version 3. Domains are color coded as indicated in (A). Residues p.R982, p.G1048, and p.E1136 are labeled with pink spheres and text box. Front view and side view of the protein is given on top and bottom, respectively. Structures were also predicted for mutants p.R982C, p.E1136Q, and p.G1048R. In (E–G), predicted structures are shown for WT *SAMD9* (left) and mutant *SAMD9* (right). Stars highlight the site of the associated mutations, predicted hydrogen bonds are indicated with yellow dashes, and interacting residues are shown with sticks. (E) Predicted structure of WT and mutant p.R982C *SAMD9*, zoomed to show residue 982. (F) Predicted structure of WT and mutant p.E1136 *SAMD9*, zoomed to show residue 1136. (G) Predicted structure of WT and mutant p.G1048R *SAMD9*, zoomed to show residue 1048. Structure visualizations were made in Pymol v2.3.4. Graphs were made in Prism Graphpad v10.2.3.

MIRAGE is an acronym for a multi‐organ syndrome of myelodysplasia, infection, restriction of growth, adrenal hyperplasia, genital phenotypes, and enteropathy caused by gain‐of‐function (GOF) mutations in *SAMD9* which most frequently results in adrenal insufficiency and inherited bone marrow failure (IBMF). *SAMD9* contains conserved functional protein domains which include a protein interacting SAM domain, a nucleic acid binding DBD domain, a putative P‐loop NTPase domain, a tandem TPR repeat domain, and a DNA binding OB domain [1, 2] (Figure 1A). *SAMD9* functions as pox‐virus restriction factors and displays inducible expression by type 1 or type 2 interferons [6, 7, 8] following inflammatory stimuli. Indeed, the promoter of *SAMD9* contains a predicted IRF1 binding site and an IFNγ response element (IRE) [9]. SAMD9 is subsequently activated through detection of viral pathogen‐associated molecular patterns (PAMPs), potentiating its function as an anticodon nuclease which cleaves phenylalanine bound tRNAs through its DBD domain, resulting in pre‐polysome ribosomal stalling, impairment of global translation, proteotoxic stress, and the suppression of viral replication [3, 4, 8]. Consistent with the presentation of MIRAGE syndrome with IBMF, *SAMD9* activation in both human [8] and murine [7, 10] models leads to deleterious effects, particularly on hematopoietic stem cells, including impaired cellular proliferation, accrual of DNA damage, and apoptosis [4, 8]. In vitro characterization of *SAMD9* GOF mutations further provides evidence of other defective processes including increased receptor internalization of EGFR, which may contribute to an impaired capacity to respond to growth factors [10, 11], and altered endo‐lysosomal function, which includes increased early endosomal fusion events [11] and lysosomal activation [10].

The expression of the profile of *SAMD9* corresponds to the clinical spectrum of organ‐specific disease manifestations in MIRAGE syndrome. *SAMD9* is highly expressed in fetal adrenal organs [2] and MIRAGE syndrome presents with primary adrenal insufficiency in roughly 78.8% of patients, although “non‐adrenal” MIRAGE syndrome patients are not infrequent and occur in 21.2% of patients [2]. *SAMD9* is also highly expressed in both primary hematopoietic (bone marrow, thymus) and secondary lymphoid organs (spleen, lymph nodes) [12], with particularly high expression within natural killer (NK) cells and neutrophils across both humans and mice [12, 13]. Consistently, *SAMD9* GOF MIRAGE syndrome patients frequently show evidence of primary immune defects including NK cell lymphopenia [14] and neutropenia [15, 16] and are prone to recurrent invasive infections [11]. Additionally, MIRAGE syndrome patients frequently present with IBMF as evidenced by hypocellularity on bone marrow biopsy [16] and concomitant thrombocytopenia and anemia [11]. In conjunction with IBMF, MIRAGE syndrome patients show an increased risk of myelodysplastic syndrome (MDS) associated with microdeletions in chromosome 7 (monosomy 7) [11], indeed 17% of pediatric patients with MDS have mutations in *SAMD9* and its paralogue *SAMD9L* [17]. Chromosome 7 contains the *SAMD9* gene loci, and deletion of the antiproliferative *SAMD9* GOF mutant results in a clonal selective advantage referred to as revertant mosaicism which mitigates bone marrow failure but precipitates hematologic malignancy [7, 18].

Given the susceptibility of MIRAGE syndrome patients to infection, the interferon inducible nature of *SAMD9*, and the survival advantage of monosomy 7 in the setting of MIRAGE syndrome, it has been speculated that recurring infections can be a driving force in promoting exacerbation of MIRAGE syndrome, subsequent revertant mosaicism, and the precipitation of monosomy 7 associated MDS [19]. Herein, we present the case of a 32‐week premature newborn female with clinical features consistent with MIRAGE syndrome associated with a previously unreported [5] *de novo* mutation, *SAMD9* G1048R, which was identified at 23 days of age by whole exome sequencing. At least 56 patients have been reported to develop either MIRAGE syndrome associated with SAMD9 mutations [5], with a median age of diagnosis at 1.18 years (Figure 1B), thus this case represents an early identification of a SAMD9 mutation associated with signs and symptoms of MIRAGE syndrome. Immunologic work‐up of our patient demonstrated minimal evidence of defects, which may reflect the phenotype spectrum of MIRAGE syndrome as well as a minimal degree of immunologic disease burden corresponding with homeostatic *SAMD9* expression prior to interferon/infection induced expression and exacerbation outside of the neonatal window.

## 2 Case History/Examination

The patient is a female who was born at 32 weeks by urgent caesarean section due to category II fetal heart tracing (FHTs) with deep decelerations in the setting of severe intrauterine growth restriction (< 1% percentile, IUGR), intermittently absent end‐diastolic flow, and fetal arrhythmia. Maternal betamethasone was initiated 2 days prior to delivery; however, the patient was initially apneic and was admitted to the neonatal intensive care unit (NICU) for respiratory management by continuous positive airway pressure (CPAP). The patient is of German, Irish, Syrian, and Iraqi descent. There was no family history of consanguinity or congenital disease. Her physical exam demonstrated down‐slanting palpebral fissures, low‐set ears, but otherwise normal facial features. Normal female genitalia were noted without evidence of virilization or malformation. Chest X‐ray (CXR) demonstrated a normal cardiac silhouette and minimal evidence of respiratory distress, and she was weaned to room air over the next week. Routine prenatal labs, newborn metabolic screen, and T‐cell receptor excision circle (TREC) screen were unremarkable.

Her presentation with IUGR and abnormal FHT was initially concerning for placental insufficiency resulting from chorioamnionitis and she was started on ampicillin and gentamicin. A sepsis work‐up was initiated which revealed negative bacterial cultures but severe thrombocytopenia to 18 k/mm^3^ on complete blood count (CBC). This raised concern for bone marrow suppression from intrauterine cytomegalovirus (CMV); however, the patient's salivary CMV testing returned negative. Blood smears demonstrated normal platelet morphology, size, and immature platelet fraction inconsistent with destructive thrombocytopenia. Her thrombocytopenia persisted, requiring multiple platelet transfusions to maintain platelet levels > 20 k/mm^3^; however, she did not go on to develop any symptoms concerning for bleeding during her hospitalization.

In the NICU she also developed recurring episodes of supraventricular tachycardia (SVT) to heart rates of 300 beats per minute, which required the use of adenosine for initial control. EKG found evidence of mild peaked T‐waves, but the echocardiogram did not reveal structural heart disease. Given her prematurity and SVTs, a complete metabolic panel was obtained to evaluate for electrolyte disturbances which revealed significant hyperkalemia to 7.5 mmol/L. Additional electrolyte disturbances included hyponatremia to 131 mmol/L, hypochloremia to 92 mmol/L, and low bicarbonate to 19 mmol/L. Her hyperkalemia was controlled with treatment by calcium gluconate, albuterol, furosemide, insulin, and D10 bolus. However, normalization of potassium did not resolve her recurring SVT and she was started on propranolol. Further evaluation for an underlying renal cause of her hyperkalemia (e.g., renal obstruction), by ultrasound found no underlying structural abnormalities. During this period, the patient developed recurrent hypotension to 50 s (systolic)/30 s (diastolic) and received a course of stress‐dosed steroids with hydrocortisone to provide mineralocorticoid activity and treat her hypotension.

## 3 Differential Diagnosis, Investigations, and Treatment

Additionally, the work‐up for an underlying endocrinologic etiology of her hyperkalemia like congenital adrenal hyperplasia, pseudo hypoaldosteronism, or adrenal insufficiency was initiated. Lab testing found no evidence of 17‐OHP elevation, and initially a random cortisol was not found to be normal. However, two subsequent adrenocorticotropin hormone (ACTH) stimulation tests (with 17.5 mcg and 125 mcg cosyntropin) demonstrated failure to induce cortisol secretion after 1 h, consistent with primary adrenal insufficiency. Her serum aldosterone was also found to be low at 8 mg/dL despite her hypotension. Collectively, her lab testing was consistent with primary adrenal insufficiency with glucocorticoid and aldosterone insufficiency causing hypotension, hyperkalemia, and her other metabolic disturbances. She was initiated on glucocorticoid replacement with hydrocortisone and aldosterone replacement with fludrocortisone, as well as sodium supplementation.

Given the patient's constellation of IUGR, thrombocytopenia, and hyperkalemia with evidence of adrenal insufficiency, an underlying genetic syndrome was suspected. Genetics were consulted and rapid trio (mother, father, and patient) whole exome sequencing was performed at 23 days of age, which returned a *de novo* likely pathogenic variant of unknown significance (VUS) in *SAMD9* (c.3142G>A, p.G1048R), a gene associated with MIRAGE syndrome (Figure 1A). Another paternally inherited likely pathogenic variant in *TTN*, (c.76383_76386del, p.N25462KfsTer4) associated with dilated cardiomyopathy was also observed.

The majority of the known *SAMD9* mutations that cause MIRAGE syndrome are within the C‐terminal half of the protein (Figure 1C), and prior studies show that C‐terminal truncations of *SAMD9L* are constitutively active [3]. While the mechanism of how *SAMD9* GOF mutations promote constitutive activity remains unknown, it has been postulated that the C‐terminal half of *SAMD9* may be auto‐inhibitory until it interacts with pox‐viral PAMPs, as this type of activation mechanism is shared by other STAND protein family members [1, 3]. Consistently, the patient's *SAMD9* G1048R mutation occurs in the C‐terminal half of *SAMD9* within the tandem TPR repeat domain and is adjacent by amino acid position to other MIRAGE syndrome *SAMD9* mutants that are known to be GOF like *SAMD9* R982C and E1136Q (Figure 1A).

Although there are phenotypic similarities between this patient's presentation and other patients with MIRAGE syndrome, we do not provide in this case report functional data to validate *SAMD9* G1048R as a GOF variant. However, to understand the potential impact of this VUS, we utilized AlphaFold to predict the structure of wild‐type (WT) SAMD9 (Figure 1D) and examined the perturbations that would be introduced by similar known SAMD9 GOF mutations (R982C, E1136Q) and the VUS (G1048R) (Figure 1E–G). Doing so, we see that the G1048 residue which is mutated in this patient is also close in physical proximity to the residues where known SAMD9 GOF mutations occur—R982C and E1136 (Figure 1D). The known MIRAGE syndrome causing SAMD9 variant R982C is a mutation from arginine, a large positively charged polar residue, to cysteine, a smaller polar residue with neutral charge. Our model predicts that the R982 residue can form 7 potential hydrogen bond contacts with residues G686, D930, I984, I968, I983, and T970, and upon mutation to R982C, only the backbone hydrogen bond between R982C and I968 remains possible (Figure 1E). Similarly, the E1136Q is a mutation from glutamic acid, containing a negatively charged polar side chain to glutamine, containing an uncharged polar side chain. Our model predicts that the E1136 residue can form four potential hydrogen bond contacts with residues R1132 and W1133. The E1136Q mutant is predicted to result in the abolition of 2 hydrogen side chain bonds with R1132 (Figure 1F). These results are consistent with the R982C and E1136Q mutations leading to altered dynamics which correlate with the marked GOF demonstrated in these variants [4]. Lastly, the patient's G1048R mutation is from glycine, a small nonpolar residue, to arginine, a large positively charged polar residue. Our model predicts that G1048 forms a potential hydrogen bond contact with residue E1046. Mutation to G1048R is predicted to not only create a new set of potential hydrogen bond contacts with residues E1042 and E1028 but also displace a flexible loop within the TPR domain (Figure 1G). We speculate that these structural alterations in the SAMD9 G1048R mutation will cause disruptions akin to the R982C and E1136Q mutants which result in constitutive activity.

Due to concern for immune deficiency arising from MIRAGE syndrome, an immunologic work‐up was initiated at 4 weeks of age which revealed overall normal immunologic function and composition, including a normal absolute lymphocyte count of 5190 cells/mcL and normal B, T and NK cell subset percentages. C‐reactive protein (CRP) was within normal limits. Initial work‐up demonstrated normal levels of serum IgM and IgA. Serum IgG antibodies detected in the patient largely reflect maternal placentally transferred antibodies which diminished over time, decreasing from 218 mg/dL measured at 4 weeks of age to 85 mg/dL measured at 8 weeks of age. On subsequent evaluation at 8 weeks, a transient neutropenia of 950 cells/mcL was noted by CBC. Peripheral blood smears at this time demonstrated minimal abnormalities. The patient was able to tolerate her routine 2‐month vaccines without complications; however, she did not receive her rotavirus vaccination at that time.

The patient was further evaluated by hematology–oncology for concern of revertant mosaicism to monosomy 7 and subsequent potentiation of MDS in the setting of suspected MIRAGE syndrome. Lab testing at 6 weeks of age did not find evidence of monosomy 7 by fluorescence in situ hybridization (FISH) or any other evidence of hematologic malignancy by single nucleotide polymorphism (SNP) based microarray and leukemia gene panel next‐generation sequencing. She was ultimately discharged from the hospital at 9 weeks of age with a plan for close outpatient follow‐up with immunology and hematology–oncology.

## 4 Conclusions and Results

We present the case of a 32‐week premature female with IUGR who, following delivery, developed a constellation of persistent thrombocytopenia, hyperkalemia, and hypotension with ACTH stimulation and serum aldosterone levels supporting a diagnosis of primary adrenal insufficiency. Rapid trio whole exome sequencing performed within the neonatal period at 23 days of age demonstrated a previously undescribed *de novo SAMD9* G1048R missense mutation, consistent with an underlying diagnosis of MIRAGE syndrome. While the patient presented with several key features of MIRAGE syndrome, namely IUGR, primary adrenal insufficiency, and thrombocytopenia, she lacked other features of the disease and demonstrated normal genitals and normal bowel function. Furthermore, this patient has demonstrated overall normal markers of immune function and has yet to develop signs or sequelae of immune deficiency and bone marrow failure typical of MIRAGE syndrome including severe infection and myelodysplasia. This may reflect either the clinical spectrum of MIRAGE syndrome [2] or the inducible nature of *SAMD9* as an ISG [6, 9].

The SAMD9 mutation in this patient, G1048R, has not previously been reported in the literature, and structural analysis using predicted tertiary structures suggests that the residue mutated in the G1048R VUS is in close physical proximity to other defined MIRAGE syndrome‐causing SAMD9 mutants including R982C and E1136Q. Our structural analysis further predicts that the G1048R, R982C and E1136Q mutations all result in disruptions between potential hydrogen bond contacts present in nonmutant SAMD9, which are predicted to result in altered domain dynamics that may associate with constitutive activity. Additionally, we also observe that the G1048R mutation is predicted to promote structural displacement within a TPR domain loop, as another potential source of altered protein dynamics. Thus, while our report lacks functional data to substantiate the *SAMD9* G1048R variant seen in this patient as GOF, our structural analysis and the patient's presentation with several features of MIRAGE syndrome are consistent with *SAMD9* G1048R representing a GOF variant.

## 5 Discussion

**FIGURE 2:**
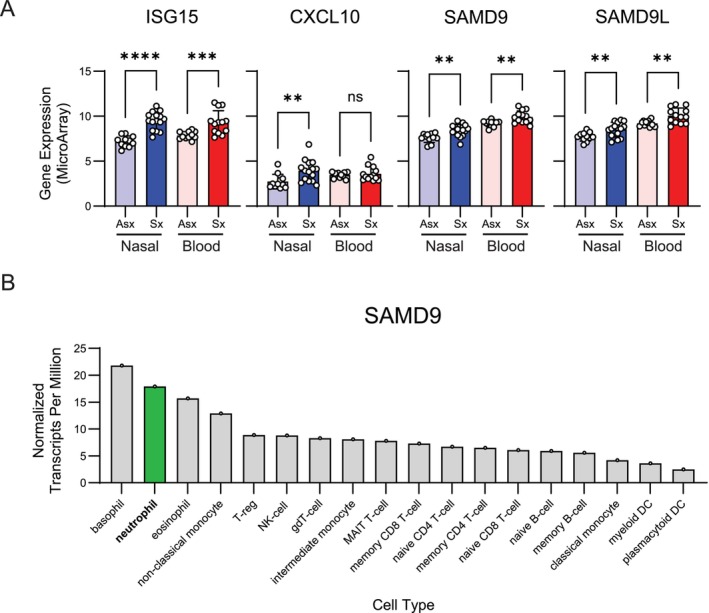
*SAMD9* and *SAMD9L* expression is elevated in symptomatic viral infections and is highly expressed in neutrophils. (A) Gene expression data for ISGs ISG15, CXCL10, SAMD9, and SAMD9L were extracted from RNA microarray data (GSE117827 [21]) generated using nasal swab (blue) and blood (red) of asymptomatic pediatric patients (Asx, *n* = 11) or symptomatic pediatric patients with acute respiratory illness and positive PCR testing for a respiratory viral pathogen (Sx, *n* = 15). One‐way ANOVA testing was used to determine statistical significance comparing nasal and blood derived samples from asymptomatic and symptomatic patients. ns, not significant; *p* > 0.05; **p < 0.01; ***p < 0.001; ****p < 0.0001. (B) SAMD9 transcript expression across 18 immune cell‐types in human peripheral blood mononuclear cells determined from scRNAseq and obtained from the Human Protein Atlas [12]. Transcript expression is reported as a normalized transcript per million. Neutrophils are labeled in green. Graphs were made and statistical analysis were performed in Prism Graphpad v10.2.3.

MIRAGE syndrome arising from *SAMD9* mutations can have a spectrum of clinical severity [2, 5]; however, given the early identification of the *SAMD9* mutation in this patient, we suspect that the absence of myelodysplasia and the absence of immune deficiency may represent low‐grade disease owing to homeostatic *SAMD9* expression prior to IFN‐induced expression of *SAMD9* following accumulation of challenges like infection over the course of early life. Indeed, interferon‐stimulated genes (ISGs) including *CXCL10*, *ISG15*, *SAMD9* and *SAMD9L* are elevated in nasal and blood samples from pediatric patients with acute respiratory symptoms and detection of respiratory viruses as compared to asymptomatic controls [20, 21] (Figure 2A). Consistently, the immune defects that we noted in this patient reflected immune cell types demonstrated to express relatively high levels of homeostatic *SAMD9*, particularly neutrophils, with this patient having developed a single episode of mild transient neutropenia [12, 13] (Figure 2B). The risk for developing immune deficiency may continue to increase as this patient grows older, develops waning of maternally derived antibodies, and is exposed to common pathogens. Thus, surveillance testing of markers of immunologic function like serum immunoglobulin levels, CBC, and lymphocyte subsetting will serve as important indicators of future decline in immunologic function and susceptibility to severe infections.

Similarly, given the risk for myelodysplastic syndrome in the setting of suspected MIRAGE syndrome, this patient will undergo surveillance testing for clonal hematopoiesis, with consideration for bone marrow transplantation if needed for the treatment of myelodysplastic syndrome. A baseline bone marrow biopsy is expected to occur around 6 months of age. However, although many of the defects associated with immunologic dysfunction and hematologic risk in MIRAGE syndrome are attributable to hematopoietic cell intrinsic defects, bone marrow transplants have been reported to be challenging in MIRAGE syndrome patients and are frequently complicated by poor outcomes related to the risk of overwhelming infection [16].

Treatment of MIRAGE syndrome patients with intravenous immunoglobulins (IVIG) has been reported to promote clinical improvement, potentially resulting from the immunomodulatory effects of IVIG and/or the immune‐protective effects of pooled immunoglobulin against environmental pathogens [22]. Noting this evidence and the patient's decreasing levels of serum IgG to 85 mg/dL at 8 weeks of age, she received and tolerated IgG repletion with IVIG (2 g/kg). Subsequent IgG studies showed normalization of serum IgG to 778 mg/dL at 11 weeks of age. Reassuringly, her serum IgA and IgM levels at this time remained normal at 13 and 117 mg/dL, respectively.

The identification of this patient with a *SAMD9* G1048R VUS at an early age represents an opportunity to better understand the early natural progression of MIRAGE syndrome and to initiate early interventions with recently reported therapies like IVIG for the treatment of more significant immunologic complications of MIRAGE syndrome as needed. As rapid genetic testing facilitates earlier identification of patients with MIRAGE syndrome and other rare genetic disorders, we are hopeful that this will facilitate both a deeper understanding of the disease, increased surveillance, and early interventions which can mitigate complications and enhance patient quality of life.

## Author Contributions

**Kevin MingJie Gao:** conceptualization, data curation, formal analysis, investigation, methodology, visualization, writing – original draft, writing – review and editing. **Qiu Yu Judy Huang:** formal analysis, writing – review and editing. **Polly Huang:** supervision, writing – review and editing. **Anum Muzaffar:** writing – review and editing. **Ramin Beheshti:** data curation, writing – review and editing. **Robert Wood:** supervision, writing – review and editing. **Jennifer Dantzer:** conceptualization, project administration, supervision, writing – review and editing. **Maria J. Gutierrez:** conceptualization, project administration, supervision, writing – review and editing.

## Ethics Statement

Ethics approval and consent to participate were obtained from all parties involved prior to publication.

## Consent

Written informed consent was obtained from the patient's family to publish this case report.

## Conflicts of Interest

The authors declare no conflicts of interest.

## Data Availability

The authors have nothing to report.
